# ATP-sensitive potassium channels gene polymorphism rs1799858 affects the risk of macro-/micro-vascular arteriosclerotic event in patients with increased low-density lipoprotein cholesterol levels

**DOI:** 10.1186/s12944-020-01315-6

**Published:** 2020-06-23

**Authors:** Cheng Liu, Tianwang Guan, Yanxian Lai, Jieming Zhu, Jian Kuang, Yan Shen

**Affiliations:** 1 Department of Cardiology, Guangzhou First People’s Hospital, School of Medicine, South China University of Technology, 1 Panfu road, Guangzhou, 510180 China; 2 Department of Cardiology, Guangzhou First People’s Hospital, Guangzhou Medical University, Guangzhou, 510180 China; 3 Department of Cardiology, the Third Affiliated Hospital, Sun Yat-sen University, Guangzhou, 510630 China; 4 Department of Cardiology, the First Affiliated Hospital, Sun Yat-sen University, Guangzhou, 510080 China

**Keywords:** ATP-sensitive potassium channels, Polymorphism, Low-density lipoprotein cholesterol, Myocardial infarction, Microalbumin in urine

## Abstract

**Background:**

Plasma concentration of low-density lipoprotein cholesterol (LDL-C) is causally related to the risk of arteriosclerotic events. Whether ATP-sensitive potassium channels (*KATP*) genetic variants predict increased LDL-C concentration (≥1.8 mmol/L) and risk of macro-/micro-vascular arteriosclerotic event remain elusive.

**Methods:**

A total of 320 subjects with increased LDL-C concentration (≥1.8 mmol/L) and 320 counterpart subjects (< 1.8 mmol/L) from the South China were enrolled in this study. Three *KATP* polymorphisms (rs1799858, rs4148671 and rs78148713) were genotyped by the Sequenom MassARRAY system. Binary logistic regression analysis was used to evaluate the association of the 3 *KATP* variants with increased LDL-C concentration and carotid artery stenosis (CAS) ≥50%. Two-way ANOVA was used to analyze the association of the 3 *KATP* variants with microalbumin in urine (MAU) and high-sensitivity C-reactive protein (HsCRP) levels. Cox proportional hazards regression analysis was used to retrospectively analyse the association of the optimal variant with the risk of new onset/recurrent acute myocardial infarction (AMI).

**Results:**

Among the 3 studied *KATP* gene single nucleotide polymorphisms (SNPs), only rs1799858 (TT + CT genotype) was associated with elevated risk of LDL-C ≥ 1.8 mmol/L (adjusted OR = 2.25, 95% CI: 1.31–3.85, *P* = 0.003) and CAS ≥50% (adjusted OR = 2.80, 95% CI: 1.12–6.98, *P* = 0.028). *KATP* SNP rs1799858 was also associated with increased MAU (*P* = 0.013) and HsCRP (*P* = 0.027) levels. The follow-up for an average of 51.1-months revealed that participants carrying the T-allele (TT + CT) of rs1799858 was associated with high risk of new onset/recurrent AMI (adjusted HR = 2.90, 95% CI: 1.06–7.94, *P* = 0.038).

**Conclusion:**

The *KATP* SNP rs1799858 may be an optimal genetic predisposition marker for increased LDL-C concentration (≥1.8 mmol/L) and its related macro-/micro-vascular arteriosclerotic event risk. The KATP variant rs1799858 was associated with higher risk of macro-/micro-vascular arteriosclerotic events in patients with elevated serum LDL-C levels.

## Background

The low-density lipoprotein cholesterol (LDL-C) is considered “bad” cholesterol because an increase in its plasma concentration is an independent risk factor for atherosclerotic cardiovascular events, which is still a major global health problem [1]. Decreased LDL-C level was significantly associated with decreased risk for macro-/micro-vascular arteriosclerotic events. A couple of the methods such as dietary control, bowel surgery and lipid-lowering drugs (especially statins) are currently used for reducing LDL-C level. However, despite achieving both a lower target of < 1.8 mmol/L and an LDL-C reduction of at least 50% from the currently recommended baseline (LDL-C is between 1.8 and 3.5 mmol/L) [2], high residual risk remains in patients receiving intensive statin therapies [3]. The Mendelian randomization showed that moderate decrease in LDL-C levels (1.0 mmol/L) prevent the development of cardiovascular disease during up to 30 years of follow-up, but it is also affected by mutations in lipid metabolism-related genes [4]. These suggest that statin therapy decreases the incidence of cardiovascular events, but the benefits may be dampened or offset by high genetic risks [5]. Thus, early genetic risk identification of populations at higher risk of increased LDL-C levels will provide a possible measure for early prevention from arteriosclerotic events.

Many candidate genes have been implicated in predisposition to increased levels of LDL-C. In recent years genes of the ATP-sensitive potassium channels (*KATP*) have received lots of attention. *KATP* is a type of potassium channel, which are hetero-octameric protein complexes comprising 4 pore-forming subunits (inwardly-rectifying K^+^ channel, Kir6.x) and 4 regulatory subunits (sulfonylurea receptor, SURx), and are widely present in the heart, skeletal muscles, vascular smooth muscles, brain and pancreatic β-cells. *KATP* are not only critical high-fidelity metabolic sensors but also key end effectors of ischemic protection (especially in cardiac and cerebral ischemia) [6]. Hence, *KATP*, as gated channels coupling metabolism with ischemic protection, have become a promising new prevention and treatment target for atherosclerotic cardiovascular disease (ASCVD). The obligate octameric arrangement result from coregulation of expression on inward-rectifier potassium channel (Kir6.x) subunits (Kir6.1 and Kir6.2, encoded respectively by KCNJ8 and KCNJ11) and sulfonylurea receptor (SURx) subunits (SUR1 and SUR2, encoded respectively by ABCC8 and ABCC9) [7]. KCNJ8 and ABCC9 are immediately adjacent to one another on human chromosome 12p12.1, whereas KCNJ11 and ABCC8 are directly adjacent to each other on human chromosome 11p15.1. The *KATP* subunit constitution may change with different physiological or pathological conditions, resulting from alternative splicing of the 4 coding genes, which produce different subunits that varies in function under different conditions. In rat model the increased plasma triglyceride (TRIG) levels were reduced by a *KATP* opener [8], and high-fat diet-feeding induced elevation in plasma TRIG and TRIG-rich very-low-density lipoproteins were restored by direct activation of *KATP* via the hepatic vagus [9]. ATP-binding cassette transporter A1 (ABCA1) is the key regulator of high-density lipoprotein cholesterol (HDL-C) [10] and apolipoprotein AI (Apo AI) [11] metabolism, while the SUR1 subunit is the same structure as ABCA1 and belongs to ATP-binding cassette superfamily, which has similar functions to ABCA1 in regulating HDL-C metabolism [10]. On the other hand, Ling et al. [12] found that the up-regulation of *KATP* in monocytes/macrophages correlated with increased inflammation in vulnerable plaques. Fan et al. [13] also found that smoking induced atherosclerosis was rescued by the inhibition of *KATP*. In rat model of myocardial infarction (MI) the cardiac protective effect of dodecafluoropentane emulsion was shown to be *KATP*-dependent [14], and the *KATP* activation by nicorandil significantly reduced the incidence of all cardiovascular events (including cardiovascular death, non-fatal MI, non-fatal stroke, or unplanned hospital admission for cardiac chest pain, etc) by 14% in patients with stable angina during 1.6-year follow up [15]. In a rat model of ischemic stroke there was larger infarct areas and more severe brain edema and neurological deficits due to astrocytic Kir6.1 knockout [16]. These suggest that *KATP* is an important link in the development from dyslipidemia to macro-/micro-vascular arteriosclerotic diseases.

Importantly, the *KATP* gene exhibits characteristics with high level of genetic polymorphism. *KATP* polymorphisms not only associated with dyslipidemia (e.g., higher levels of triglyceride (TIRG), total cholesterol (TC) and LDL-C as well as lower HDL-C) [17–19], essential hypertension (EH) [20] and ASCVD (e.g., MI and stroke) [21, 22] but also exhibited geographical and ethnic diversity (e.g., Africans, Europeans, East Asians). However, the relationship of *KATP* single nucleotide polymorphisms (SNPs) with increased LDL-C levels and its related macro-/micro-vascular arteriosclerotic events in China is rarely reported. Theoretically, there is a common genetic basis for dyslipidemia, EH and its related atherosclerotic cardiovascular events [23, 24]. The present study was undertaken to investigate the possible relationships of *KATP* gene SNPs with the risk of increased LDL-C levels (≥1.8 mmol/L) and macro-/micro-vascular arteriosclerotic events in South China.

## Materials and methods

### Study participants

This study was by the Ethics Committee of Guangzhou First People’s Hospital, School of Medicine, South China University of Technology (K-2017-043-02). A total of 320 subjects with increased LDL-C level (≥1.8 mmol/L) and 320 counterpart subjects (< 1.8 mmol/L) from the South China were enrolled in the study. All participants with different types of dyslipidemia were newly diagnosed according to guidelines [2], including high levels of LDL-C (≥1.8 mmol/L), TRIG (≥1.7 mmol/L), TC (≥5.2 mmol/L) or (and) apolipoprotein B (Apo B ≥ 80 mg/dL), and (or) low levels of HDL-C (< 1.0 mmol/L) and Apo AI (< 120 mg/dL). All participants with the coexisting EH, coronary atherosclerotic heart disease (CAD), type 2 diabetes mellitus (T2D) or (and) atrial fibrillation (AF) were also recently diagnosed according to relevant guidelines (details guidelines information was presented in methods section of Additional file 1). Clinical data were collected from patient interviews, review of medical records and contact with treating physicians. All blood biochemistry analysis was conducted on admission to the study by using standard analytical techniques. Cardiac and bilateral carotid ultrasonography were performed on admission to the study according to the recommendations from the American Society of Echocardiography [25] and the North American Symptomatic Carotid Endarterectomy Trial [26], respectively.

### Genotyping assay

Three *KATP* SNPs (rs1799858, rs4148671 and rs78148713) were genotyped using the MassARRAY system (Sequenom) according to the manufacturer’s instructions with minor modifications based on literatures and human genome sequence databases [27]. Primers for the 3 *KATP* SNPs were designed based upon sequence information from GenBank using Primer 5.0 (Whitehead Institute Cambridge, Massachusetts, USA) according to the *KATP* gene sequence in GenBank [NC_000011.10, NC_000012.12 and NC_000012.11 (Additional file 1: Table S1)]. The primers were synthesized by Invitrogen Life Science Technologies (Guangzhou, China). The accuracy of the genotypes determined was 100% for each *KATP* SNP as described above.

### Long-term follow-up endpoint

Primary outcome was new onset/recurrent acute myocardial infarction (AMI) including ST segment elevation MI and non ST segment elevation MI [28], and was diagnosed according to the guidelines at the time. The following three types of information sources were used to identify the primary outcome: participants and their families, medical records and the Center for Disease Control and Prevention. Patients were enrolled in the study from the date of first diagnosis of dyslipidemia, EH, CAD, T2D or (and) AF. Moreover, patient’s AMI-free event survival time was calculated from the date of entry to the date of first diagnosis AMI, recurrent AMI or last follow-up. The date of final follow-up was December 31, 2019.

### Statistical analysis

All analysis was using SPSS version 24 (SPSS, Chicago, IL). The Hardy-Weinberg equilibrium was assessed with a Chi-square test for control subjects as shown in Additional file 1: Table S2. Categorical variables [gender, smoking, drinking, EH, CAD, T2D, AF, high LDL-C (≥1.8 mmol/L), high TRIG (≥1.7 mmol/L), high TC (≥5.2 mmol/L), low HDL-C (< 1.0 mmol/L), high Apo B (≥80 mg/dL), low Apo AI (< 120 mg/dL), carotid artery stenosis (CAS) ≥50% and new-onset/recurrent AMI] were presented as frequencies. The associations between each *KATP* SNP and these categorical variables were analyzed using the Chi square test. Binary logistic regression analysis was used to evaluate the Odds ratio (OR) of these types of dyslipidemia and CAS ≥50%. Given that false positive might exist in the results, Bonferroni adjustment was used to adjust the significance thresholds for multinomial logistic regression. The Cox proportional hazards regression analysis for event free analysis of new onset/recurrent AMI was used to estimate the crude and adjusted hazard ratios (HRs) as well as the 95% confidence intervals (CIs) with adjustments for potential covariates. Continuous variables were presented as mean ± SD. The differences between continuous variables between the two groups were evaluated by two-way ANOVA or independent-sample t-test. *P* values of less than 0.05 were considered as statistically significant. All probabilities are two-tailed estimated.

## Result

### Characteristics of the study participants

As shown in Table 1, participants with or without increased LDL-C level (≥1.8 mmol/L) showed significant differences in the levels of TRIG (*P* = 0.003), TC (*P* < 0.001), Apo B (*P* < 0.001), microalbumin in urine (MAU, *P* < 0.001) and high-sensitivity C-reactive protein (HsCRP, *P* = 0.009).

**Table 1:** Baseline characteristics of study participants

	LDL-C < 1.8 mmol/L	LDL-C ≥ 1.8 mmol/L	***P*** value
N	320	320	–
Male: Female	232:88	217:103	0.195
Age (Y)	65.2 ± 11.3	65.5 ± 11.1	0.748
Smoking (%)	143 (44.7)	150 (46.9)	0.579
Drinking (%)	40 (12.5)	44 (13.8)	0.640
SBP (mmHg)	139.4 ± 21.2	142.4 ± 23.8	0.098
DBP (mmHg)	77.4 ± 13.1	78.5 ± 13.0	0.278
BMI (kg/m^2^)	24.5 ± 4.2	24.8 ± 4.3	0.394
**Medical condition**			
EH (%)	214 (66.9)	236 (73.8)	0.057
CAD (%)	266 (83.1)	255 (79.7)	0.264
T2D (%)	174 (54.4)	174 (54.4)	1.000
AF (%)	10 (3.1)	17 (5.3)	0.169
**Blood biochemical index**			
TRIG (mmol/L)	1.46 ± 1.01	1.70 ± 1.00	0.003
TC (mmol/L)	3.27 ± 0.70	4.70 ± 1.12	< 0.001
HDL-C (mmol/L)	1.07 ± 0.29	1.08 ± 0.23	0.507
Apo B (mg/dL)	64.9 ± 21.4	100.4 ± 33.0	< 0.001
Apo A1 (mg/dL)	102.4 ± 23.7	103.4 ± 23.3	0.560
WBC (×10^9^/L)	8.31 ± 2.90	8.32 ± 2.61	0.962
HGB (g/L)	132.3 ± 18.8	129.8 ± 17.2	0.076
PLT (×10^9^/L)	228.6 ± 64.0	237.4 ± 66.3	0.088
FBG (mmol/L)	5.48 ± 1.20	5.62 ± 1.48	0.173
P2hBS (mmol/L)	9.01 ± 2.71	8.96 ± 2.66	0.825
HbA1C (%)	6.0 ± 1.1	6.0 ± 1.3	0.926
Scr (μmol/L)	92.0 ± 46.9	89.7 ± 34.0	0.468
BUN (mmol/L)	5.72 ± 2.09	5.81 ± 1.97	0.569
UA (μmol/L)	402.1 ± 107.8	397.4 ± 104.6	0.573
ALT (U/L)	25.7 ± 24.3	27.3 ± 34.4	0.483
AST (U/L)	47.9 ± 112.8	52.3 ± 105.4	0.612
Alb (g/L)	37.2 ± 3.5	36.7 ± 4.9	0.104
Na^+^ (mmol/L)	140.3 ± 3.4	140.2 ± 3.2	0.613
K^+^ (mmol/L)	3.76 ± 0.38	3.74 ± 0.43	0.416
HsCRP (mg/L)	10.9 ± 11.1	14.2 ± 19.1	0.009
MAU (ACR, mg/g)	229.0 ± 392.2	355.1 ± 480.2	< 0.001
HCY (μmol/L)	14.7 ± 5.7	14.2 ± 5.9	0.342
ACE (U/L)	35.1 ± 23.2	33.6 ± 20.1	0.380
Renin (pg/mL)	27.1 ± 28.5	25.5 ± 27.2	0.479
Ang I (ng/L)	2.40 ± 1.79	2.51 ± 1.38	0.427
Ang II (ng/L)	64.4 ± 85.0	58.0 ± 78.9	0.325
ALD (ng/L)	171.0 ± 120.1	170.2 ± 88.3	0.928
**Echocardiography**			
**RVD (cm)**	1.73 ± 0.19	1.75 ± 0.17	0.296
**RAD (cm)**	3.36 ± 0.30	3.31 ± 0.30	0.064
**LVD (cm)**	4.83 ± 0.58	4.76 ± 0.54	0.095
**LAD (cm)**	3.11 ± 0.56	3.07 ± 0.57	0.336
**LVEF (%)**	55.9 ± 10.8	56.7 ± 8.5	0.276

### Association between *KATP* SNPs and elevated LDL-C (≥1.8 mmol/L)

As shown in Table 2, *KATP* SNP rs1799858 (TT + CT genotype) was associated with elevated risk of high LDL-C (≥1.8 mmol/L) levels (adjusted OR = 2.25, 95% CI: 1.31–3.85, *P* = 0.003).

**Table 2:** Association between *KATP* SNPs and elevated LDL-C (≥1.8 mmol/L) in study participants

***KATP*** SNPs		LDL-C ≥ 1.8 mmol/L (N/%)		Crude OR (95% CI)	Crude ***P*** value	Adjusted OR (95% CI)^a^	Adjusted ***P*** value^a^	Adjusted OR (95% CI)^**b**^	Adjusted ***P*** value^**b**^
		NO	YES						
*rs1799858*	*CC*	218 (68.1)	202 (63.1)	1.00		1.00		1.00	
	*TT + CT*	102 (31.9)	118 (36.9)	1.25 (0.90–1.73)	0.183	1.50 (1.04–2.16)	0.032	2.25 (1.31–3.85)	0.003
*rs4148671*	*CC*	276 (86.3)	288 (90.0)	1.00		1.00		1.00	
	*TT + CT*	44 (13.8)	32 (10.0)	0.70 (0.43–1.13)	0.144	0.72 (0.42–1.23)	0.224	0.61 (0.35–1.06)	0.078
*rs78148713*	*CC + CT*	20 (6.3)	12 (3.8)	0.58 (0.28–1.22)	0.151	0.51 (0.24–1.11)	0.091	0.96 (0.34–2.72)	0.935
	*TT*	300 (93.7)	308 (96.2)	1.00		1.00		1.00	

^a^Model 1: After adjustment for gender, age, smoking, drinking, WBC, BMI, EH, T2D, liver function (ALT, AST and Alb), renal function (Scr, BUN and UA), HsCRP, HbA1C, HCY, and RAAS activity (ACE, renin, Ang I, Ang II and ALD)

^b^Model 2: It is the same as Model 1, and also including dyslipidemia (TRIG, TC, Apo B, HDL-C and Apo AI)

### Association between *KATP* SNPs and CAS ≥50%

As shown in Table 3, *KATP* SNP rs1799858 (TT + CT genotype) was associated with increased risk of CAS ≥50% (adjusted OR = 2.80, 95% CI: 1.12–6.98, *P* = 0.028).

**Table 3:** Association between *KATP* SNPs and CAS ≥50% in study participants

***KATP*** SNPs		CAS ≥ 50% (N/%)		Crude OR (95% CI)	Crude ***P*** value	Adjusted OR (95% CI)^a^	Adjusted ***P*** value^a^	Adjusted OR (95% CI)^**b**^	Adjusted ***P*** value^**b**^
		NO	YES						
*rs1799858*	*CC*	408 (66.6)	12 (44.4)	1.00		1.00		1.00	
	*TT + CT*	205 (33.4)	15 (55.6)	2.49 (1.14–5.41)	0.022	2.74 (1.09–6.88)	0.032	2.80 (1.12–6.98)	0.028
*rs4148671*	*CC*	542 (88.4)	22 (81.5)	1.00		1.00		1.00	
	*TT + CT*	71 (11.6)	5 (18.5)	1.74 (0.64–4.73)	0.281	2.35 (0.69–8.15)	0.178	2.60 (0.74–9.12)	0.136
*rs78148713*	*CC + CT*	30 (4.9)	2 (7.4)	1.56 (0.35–6.87)	0.561	1.26 (0.24–6.59)	0.782	1.34 (0.25–7.04)	0.734
	*TT*	583 (95.1)	25 (92.6)	1.00		1.00		1.00	

^a^Model 1: After adjustment for gender, age, smoking, drinking, WBC, BMI, EH, T2D, liver function (ALT, AST and Alb), renal function (Scr, BUN and UA), HsCRP, HbA1C, HCY, and RAAS activity (ACE, renin, Ang I, Ang II and ALD)

^b^Model 3: It is the same as Model 1, and also including dyslipidemia (TRIG, TC, LDL-C, Apo B, HDL-C and Apo AI)

### Association between *KATP* SNPs and MAU level

As shown in Table 4, participants with the increased LDL-C risk genotype of rs1799858 (TT + CT) was associated with elevated MAU level (*P* = 0.001 and < 0.001) whereas rs4148671 (both *P* > 0.05) and rs78148713 (both *P* > 0.05) were not significantly associated.

**Table 4:** Association between *KATP* SNPs and MAU level in participants

***KATP*** SNPs		MAU (ACR, mg/g)		
		LDL-C < 1.8 mmol/L	LDL-C ≥ 1.8 mmol/L	***P value***
*rs1799858*	*CC*	231.2 ± 440.2	286.2 ± 519.5	0.241
	*TT + CT*	224.3 ± 263.8	473.2 ± 377.7	< 0.001
	*P value*	0.885	0.001	
*rs4148671*	*CC*	211.1 ± 382.6	351.5 ± 476.5	< 0.001
	*TT + CT*	341.2 ± 435.9	388.0 ± 518.7	0.671
	*P value*	0.067	0.684	
*rs78148713*	*CC + CT*	203.4 ± 204.9	463.5 ± 552.0	0.141
	*TT*	230.7 ± 410.8	350.9 ± 477.7	0.001
	*P value*	0.764	0.426	

^a^*ACR* urinary albumin-to-creatinine ratio

### Association between *KATP* SNPs and HsCRP level

As shown in Table 5, participants with the increased LDL-C risk genotype of rs1799858 (TT + CT) was associated with elevated HsCRP level (*P* = 0.018 and 0.008) whereas rs4148671 (both *P* > 0.05) and rs78148713 (both *P* > 0.05) were not significantly associated.

**Table 5:** Association between *KATP* SNPs and HsCRP level in participants

***KATP*** SNPs		HsCRP (mg/L)		
		LDL-C < 1.8 mmol/L	LDL-C ≥ 1.8 mmol/L	***P value***
*rs1799858*	*CC*	10.7 ± 10.7	12.2 ± 17.1	0.295
	*TT + CT*	11.2 ± 11.8	17.5 ± 21.8	0.008
	*P value*	0.712	0.018	
*rs4148671*	*CC*	10.8 ± 11.3	14.0 ± 19.5	0.016
	*TT + CT*	11.5 ± 9.8	15.4 ± 15.3	0.211
	*P value*	0.677	0.688	
*rs78148713*	*CC + CT*	13.6 ± 6.7	15.5 ± 17.0	0.719
	*TT*	10.7 ± 11.3	14.1 ± 19.2	0.008
	*P value*	0.083	0.799	

### Association between *KATP* SNP rs1799858 and new-onset/recurrent AMI

**Fig. 1:**
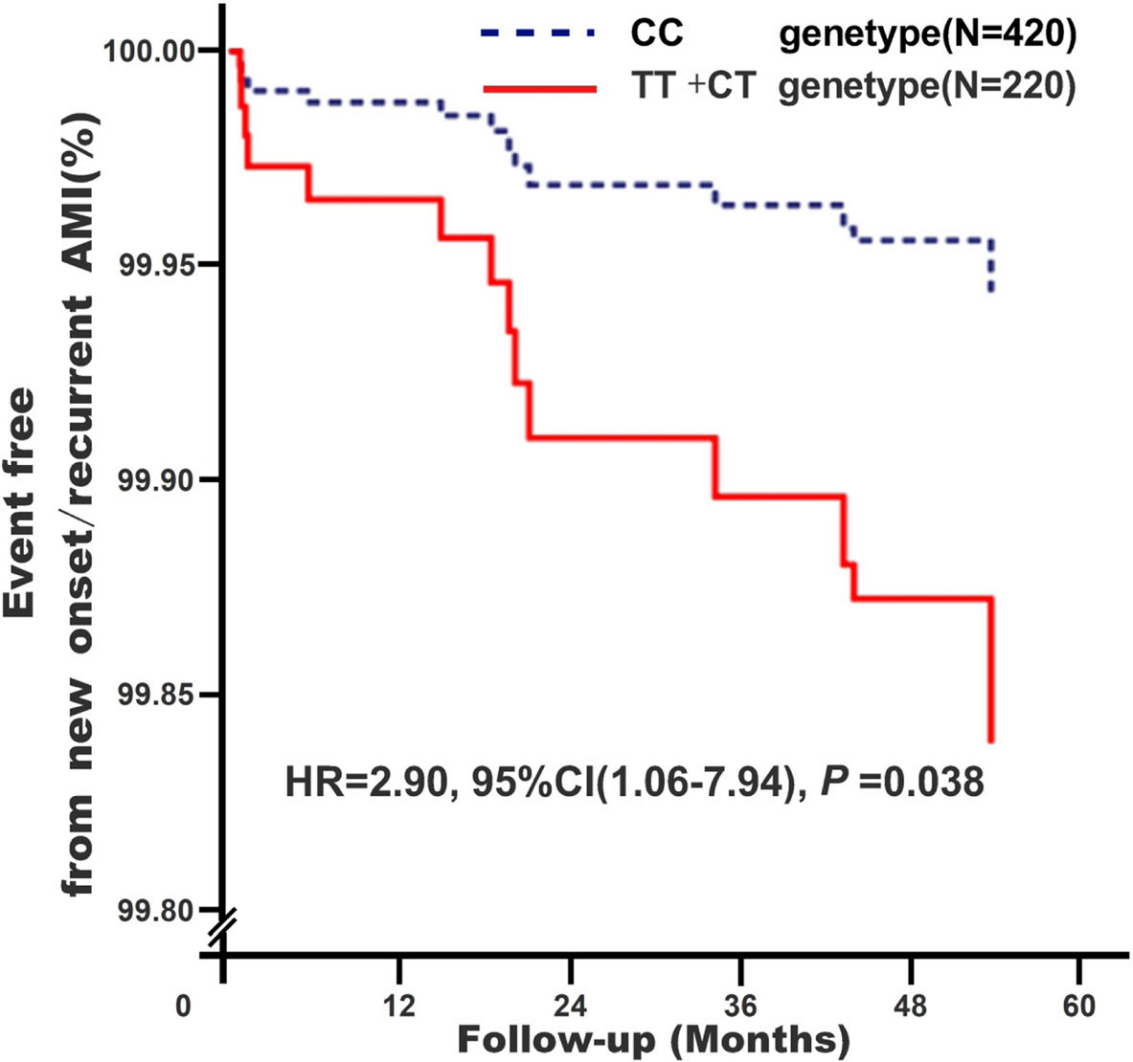
Association between KATP rs1799858 and new-onset/recurrent AMI in participants*. *Model 4: After adjustment for gender, age, smoking, drinking, WBC, BMI, liver function (ALT, AST and Alb), renal function (Scr, BUN and UA), HsCRP, HbA1C, HCY, and RAAS activity (ACE, renin, Ang I, Ang II and ALD), dyslipidemia (TRIG, TC, LDL-C, Apo B, HDL-C and Apo AI), medical condition (EH, CAD, T2D and AF), NYHA functional classification, combined medication (antiplatelet drugs, warfarin, statins, RSIs, BBs, MRA, CCBs, diuretics, digoxin, nitrates, and hypoglycemic agents) and echocardiography index (RVD, RAD, LVD, LAD, and LVEF)

As shown in Fig. 1, participants carrying T-allele (TT + CT) of rs1799858 was associated with higher risk of new-onset/recurrent AMI (adjusted HR = 2.90, 95% CI: 1.06–7.94, *P* = 0.038) during an average follow-up of 51.1-months.

## Discussion

This study is the first to address the possible associations of *KATP* gene SNPs with different types of dyslipidemia in south China. The data reported in this study indicate that none of the 3 *KATP* SNPs is associated with increased levels of TRIG (≥1.7 mmol/L, Additional file 1: Table S3), TC (≥5.2 mmol/L, Additional file 1: Table S4) and Apo B (≥80 mg/dL, Additional file 1: Table S6) as well as decreased levels of HDL-C (< 1.0 mmol/L, Additional file 1: Table S5) and Apo AI (< 120 mg/dL, Additional file 1: Table S7). Importantly, the data indicate that the risk of increase in LDL-C levels (≥1.8 mmol/L) in participants with T-allele (TT + CT) increases by 1.25-fold compared with those with CC genotype except for rs4148671 and rs78148713 variants. Dyslipidemia is a heterogeneous disease. The findings in this study are partially consistent with the study by Engwa et al. [17] reporting that the TT genotype of rs1799854 is related to high levels of HDL and low levels of TC, TG and LDL in non-diabetic patients among Nigerians, and with the study by Nikolac et al. [18] reporting that rs1799859 and rs1799854 were related to higher TRIG level in Croatia T2D patients with sulfonylurea therapy, and with the study by Xu et al. [19] reporting that the K allele (KK + EK) of rs5219 is associated with a higher ratio of TC vs. HDL-C in prediabetic youth Han Chinese.

Atherogenic dyslipidemia-characterized by preponderance of small, dense, LDL-C is the major cause of macrovascular arteriosclerotic disease. CAS ≥50% is now recognized as a new subtype of macrovascular arteriosclerotic disease besides CHD and stroke [29]. This is based on findings that the 10-year risk of ASCVD in patients with CAS ≥50% is concordant with those in patients suffering from cardiovascular disease. This study further elucidate that participants with high LDL-C risk genotype of rs11046182 (TT + CT) also associated with high risk of CAS ≥50% (increased approximately by 1.8-fold). In addition to macrovascular arteriosclerotic disease, recent studies show an association between increased LDL-C level and microvascular arteriosclerotic disease (e.g., coronary [30] and renal [31]). The MAU is a key biomarker of microvascular injury in chronic kidney disease and is an independent risk factor of cardiovascular events [32]. This study indicates an association between high LDL-C risk genotype (TT + CT) of rs1799858 and the increase in MAU levels.

Whether it is macrovascular arteriosclerotic diseases or microvascular diseases, inflammation pathways play an important role in the pathogenesis of atherosclerosis caused by abnormal LDL-C level. Recently, the Canakinumab Anti-inflammatory Thrombosis Outcome Study (CANTOS) [33] indicated that decreasing inflammation with canakinumab, an interleukin-1β neutralizing monoclonal antibody, reduced cardiovascular events independent of cholesterol level [34]. On the other hand, methotrexate (MTX) exerts anti-inflammatory effects through its intracellular metabolite (polyglutamate methotrexate) and may help in preventing atherosclerotic events. However, low-doses of MTX do not reduce inflammatory factor levels (e.g., interleukin-1β, interleukin-6, or C-reactive protein) and the risk of ASCVD among patients with stable atherosclerosis [35]. Low-doses of colchicine decrease ischemic cardiovascular events by 23% among patients with recent myocardial infarction [36]. These results suggest that it is necessary to screen appropriate populations (high inflammation risk) for receiving anti-inflammatory treatment besides the differences in anti-inflammatory drugs used. This study indicates that the high LDL-C risk genotype of rs1799858 (TT + CT) associates with increased levels of the anti-inflammation marker HsCRP. Previous studies have indicated that the HsCRP levels are not only related to cardiovascular disease independent of traditional risk factors, but also can independently predict the occurrence and recurrence of adverse cardiovascular events [37, 38]. This study is the first to report that the high risk of increased LDL-C and HsCRP related T allele (TT + CT) of rs1799858 associate with higher risk of new-onset/recurrent AMI (increased approximately by 1.9-fold) after an average follow-up of 51.1-months. These observations suggest that rs1799858 may be genetic factor that predispose patients to atherogenic dyslipidemia (increased LDL-C level) related macro-/micro-vascular arteriosclerotic event risk.

Currently, the mechanism by which *KATP* variant increases LDL-C levels and its related macro-/micro-vascular arteriosclerotic event risk remains unclear. This study reports no significant difference on SBP, BMI and postprandial two hours blood glucose (P2hBS) was observed between the two groups (Table 1), but the average levels of SBP (> 130 mmHg), BMI (> 24.0 kg/m^2^) and P2hBS (> 7.8 mmol/L) were higher than normal especially in high LDL-C (≥1.8 mmol/L) group, which was also accompanied with high TRIG, TC and Apo B levels. This suggests that participants in the high LDL-C group suffer a state of metabolic syndrome (MeS). Insulin resistance (IR) plays a primary role in the pathogenesis of MeS, leading to dyslipidemia and its related ASCVD [39]. Indeed, Wan et al. [40] reported that rs5219 is associated with IR in vitro model (L6 rat skeletal muscle cell line), and Gonen et al. [41] further reported that *KATP* SNP rs1799854, rs1799859 and rs5219 play a key role in glucose-induced insulin secretion in Turkey while such association was not seen in participants from the Caribbean harboring the rs5219 variant [42] as well as participants from Polands harboring the rs1799854 variant [22]. The effects of *KATP* gene polymorphisms (mutations or variants) on IR are not fully understood, but it is tempting to speculate that the elevated activity of *KATP* as well as in the presence of the *KATP* rs1799858 variant, which directly impact the cardiovascular system, and influences the susceptibility to dyslipidemia and its related macro-/micro-vascular arteriosclerotic damages. Such influence can be tissue specific and attributes to tissue/cell-specific *KATP* variants expressed in the heart, skeletal/smooth muscles, pancreatic β-cells and the brain. AMI is an important cardiac subtype of ASCVD. The pathophysiological essence of AMI is myocardial ischemia that is the result of coronary artery (“macrovascular”) obstruction (e.g., CAD) and coronary microvascular obstruction (e.g., coronary microvascular dysfunction (CMD)). CMD, as a new microvascular paradigm, has been widespread before obstructive CAD is present in the epicardial coronary arteries, and plays the leading role in the pathophysiology of AMI. Imbalance in lipid metabolism is one of the initiating factors of CMD. Although the mechanism underlying CMD caused by dyslipidemia (e.g., LDL-C [43]) is not fully understood, existing evidence suggests that it is associated with increased oxidative stress [44]. *KATP* is not only involved in the metabolic regulation of coronary vascular tone (e.g., coronary *KATP*) but also in mediating oxidative stress (e.g., mitochondrial *KATP*) [45, 46].

ASCVD (e.g., CAD) results from a cross-talk between environmental and genetic factors [47]. Although environmental factors (e.g., high-cholesterol diet) play a role in ASCVD, genetic factors are a major determinant of ASCVD. The genetic risk of ASCVD is conferred in part through known metabolic risk factors (e.g., dyslipidemia) [48]. ASCVD results in complex pathologies that develop over time due to genetic and environmental factors. To adapt to ever-changing gene-environment interactions, posttranslational regulations (e.g., microRNA [49]) have recently been valued as core contributors to ASCVD pathogenesis. SNPs have a huge impact on transcription, maturation and target specificity of miRNA. Hence, the mutation of the microRNA binding site in the target gene will affect the splicing efficiency and stability of mRNA (e.g., intron splicing enhancer, silencer element or conformation), regulates RAAS activity [50] and mediates cardiovascular injury, attributed to *KATP* SNPs (e.g., rs1799858). Based on the aforementioned, elucidating the specific regulatory mechanism has proven to be indispensable.

### Study strengths and limitations

The strengths of the study were that this is the first time a direct association of KATP SNPs with the risk of increased LDL-C levels and macro-/micro-vascular arteriosclerotic events, and with the inter-individual variability of inflammatory state in arteriosclerotic related diseases. Another strength of this study was that it had a follow-up time of over 51 months, and evaluated risks associated with new-onset/recurrent acute coronary syndrome. This study was also hurdled by some limitations. Firstly, since the sample size of this study is not big enough (*N* = 640), large-scale prospective studies will help to further verify those findings. Secondly, Bonferroni adjustment was used to adjust the significance thresholds, but there was still the possibility of potential false-positive results especially for future research building on this study. Therefore, results must be interpreted carefully.

## Conclusion

*KATP* SNP rs1799858 may be a potential genetic susceptibility marker for increased LCL-C level and its related macro-/micro-vascular arteriosclerotic event risk. Therefore, genetic screening at the *KATP* SNP loci could potentially improve patient outcomes through early prevention or treatment for macro-/micro-vascular arteriosclerotic diseases with a much needed large-scale prospective study.

## Supplementary information

**Additional file 1: **Additional Method and Results. **Table S1.** The primers of *KATP* SNPs in the Sequenom MassARRAY system. **Table S2.***KATP* SNPs in study participants. **Table S3.** Association of *KATP* SNPs with increased TRIG (≥ 1.7 mmol/L) levels in study subjects. **Table S4.** Association of *KATP* SNPs with increased TC (≥ 5.2 mmol/L) levels in study participants. **Table S5.** Association of *KATP* SNPs with decreased HDL-C (< 1.0 mmol/L) levels in study participants. **Table S6.** Association of *KATP* SNPs with increased Apo B (≥ 80 mg/dL) levels in study subjects. **Table S7.** Association of *KATP* SNPs with decreased Apo AI (< 120 mg/dL) levels in study participants. **Table S8.** Baseline characteristics of study participants at the end of the follow-up.

## Data Availability

The datasets used and/or analyzed in this study are available from the corresponding author on reasonable request.

## Abbreviations

ACE: Angiotensin converting enzyme
ACR: Urinary albumin-to-creatinine ratio
AF: Atrial fibrillation
Alb: Albumin
ALD: Aldosterone
ALT: Alanine aminotransferase
AMI: Acute myocardial infarction
Ang I/ II: Angiotensin I/II
Apo AI: Apolipoprotein AI
Apo B: Apolipoprotein B
ASCVD: Arteriosclerosis cardiovascular disease
AST: Aspartate aminotransferase
BMI: Body mass index
BUN: Blood urea nitrogen
CAS: Carotid artery stenosis
CAD: Coronary atherosclerotic heart disease
CI: Confidence interval
CMD: Coronary microvascular dysfunction
DBP: Diastolic blood pressure
EH: Essential hypertension
FBG: Fasting blood glucose
HbA1C: Glycosylated hemoglobin
HDL-C: High-density lipoprotein cholesterol
HGB: Hemoglobin concentration
HR: Hazard ratio
HsCRP: High-sensitivity C-reactive protein
K^+^: Serum potassium
KATP: ATP-sensitive potassium channels
Kir6.x: Inward-rectifier potassium channel
LDL-C: Low-density lipoprotein cholesterol
MAF: Minor allele frequency
MAU: Microalbumin in urine
MeS: Metabolic syndrome
MI: Myocardial infarction
MTX: Methotrexate
Na^+^: Serum sodium
OR: Odds ratio
P2hBS: Postprandial blood glucose two hours
PLT: Platelet count
RAAS: Renin-angiotensin-aldosterone system
SBP: Systolic blood pressure
Scr: Serum creatinine
SNP: Single nucleotide polymorphism
SUR: Sulfonylurea receptor
TC: Total cholesterol
T2D: Type 2 diabetes mellitus
TRIG: Triglyceride
UA: Serum uric acid
WBC: White blood cell count
