# Supplementary material for: ATP-sensitive potassium channels gene polymorphism rs1799858 affects the risk of macro-/micro-vascular arteriosclerotic event in patients with increased low-density lipoprotein cholesterol levels
Source: Lipids Health Dis. 2020 Jun 23;19:147. doi: 10.1186/s12944-020-01315-6 (PMC7313205; doi:10.1186/s12944-020-01315-6)
Supplement: Supplementary file 1 — Additional file 1: Additional Method and Results. Table S1. The primers of KATP SNPs in the Sequenom MassARRAY system. Table S2.KATP SNPs in study participants. Table S3. Association of KATP SNPs with increased TRIG (≥ 1.7 mmol/L) levels in study subjects. Table S4. Association of KATP SNPs with increased TC (≥ 5.2 mmol/L) levels in study participants. Table S5. Association of KATP SNPs with decreased HDL-C (< 1.0 mmol/L) levels in study participants. Table S6. Association of KATP SNPs with increased Apo B (≥ 80 mg/dL) levels in study subjects. Table S7. Association of KATP SNPs with decreased Apo AI (< 120 mg/dL) levels in study participants. Table S8. Baseline characteristics of study participants at the end of the follow-up. [file 12944_2020_1315_MOESM1_ESM.docx]

**Additional file 1：Additional Method and Results**

**Additional Method**

**Study participants**

This study was reviewed and approved by the Ethics Committee of Guangzhou First People’s Hospital, School of Medicine, South China University of Technology (K-2017-043-02). A total of 320 subjects with increased LDL-C levels (≥ 1.8mmol/L) and 320 counterpart subjects (<1.8mmol/L) from the South China were enrolled in the study. All participants with high LDL-C levels recently diagnosed according to guidelines [1] as well as the other types of dyslipidemia, including high levels of triglyceride (TRIG ≥1.7mmol/L), total cholesterol (TC ≥5.2mmol/L) or (and) Apo B (≥80mg/dL), and (or) low level of high-density lipoprotein cholesterol (HDL-C <1.0mmol/L) and apolipoprotein AI (Apo AI <120mg/dL). All participants with the coexisting essential hypertension (EH) [2], coronary heart disease (CHD) [3], type 2 diabetes mellitus (T2D) [4] or (and) atrial fibrillation (AF) [5] were also recently diagnosed according to relevant guidelines. Clinical data were collected from patients’ interviews, medical records and contact with treating physicians. The biochemistry tests and bilateral carotid duplex scanning were conducted when patients enrolled to the study. All biochemistry tests were performed by standard methods in the Chemical Laboratory.

**Carotid and cardiac ultrasonography**

Bilateral carotid and cardiac ultrasonic scanning was performed when patients admitted to the study. The near and far walls of bilateral common carotid artery, bifurcations, and 1 cm of the internal and external carotid arteries were scanned for the presence of carotid arteriosclerosis stenosis (CAS) ≥50% (recorded as the average of measurements by two independent experienced physicians according to the measurement of stenosis degree used in the North American Symptomatic Carotid Endarterectomy Trial [6]) with a 3/9 MHz ultra-wideband linear array transducer (iU22, Philips, NL). The left atrial end-diastolic dimension (LAD), left ventricular end-diastolic diameter (LVD), right atrial end-diastolic dimension (RAD), right ventricular end-diastolic diameter (RVD), and left ventricular ejection fraction (LVEF) were measured using M-mode or two-dimensional echocardiography in the parasternal long-axis view at the end-ventricular systole with a 1.7/3.4 MHz linear array transducer (Vivid 7, GE Healthcare, USA) over 4 cardiac cycles according to recommendations for chamber quantification from the American Society of Echocardiography [7].

**Genotyping assay**

Genomic DNA was extracted from whole blood by using the Maxwell RSC Whole blood DNA kit (Promega, Madison, WI), quantified using NanoDrop-1000 (ThermoFisher, Waltham, MA) and diluted to the concentration of 10 ng/μL. Three *KATP* SNPs (rs1799858, rs4148671 and rs78148713) were analyzed using the Sequenom MassARRAY system according to previously described methods [8] based on literatures and human genome sequence databases. Primers for *KATP* SNPs were designed according to the sequence from GenBank using Primer 5.0 (Whitehead Institute Cambridge, Massachusetts, USA) and Operon’s Oligo software 7.60 (Operon Technologies Inc., Alameda, California, USA). The primers were synthesized by Invitrogen Life Science Technologies (Guangzhou, China). Primers are shown in Table S1. Genotyping accuracy was determined by genotype concordance between duplicate samples and was 100% for each SNP.

**Results**

**Characteristics of the study participants**

As shown in Table 1, participants with or without increased LDL-C levels (≥1.8 mmol/L) showed significant differences in the levels of TRIG (*P*=0.003), TC (*P*<0.001), Apo B (*P*<0.001), HsCRP (*P*=0.009) and MAU (*P*<0.001) but not in gender, age, smoking, drinking, coexisting medical conditions (EH, CHD, T2D and AF), HDL-C, Apo AI, WBC, HGB, PLT, blood glucose (FBG, P2hBS, HbA1C), renal function (Scr, BUN, UA), liver function (ALT, AST, Alb), blood electrolytes (serum sodium and potassium), the activation of RAAS (ACE, renin, Ang I/II and ALD) and echocardiography index (RVD, RAD, LVD, LAD, and LVEF) (all *P*>0.05). After an average follow-up of 51.1-months, participants with or without increased LDL-C levels showed non-significant differences in NYHA functional classification and combined medication (antiplatelet drugs, warfarin, statins, RSIs, BBs, MRA, CCBs, diuretics, digoxin, nitrates, and hypoglycemic agents), as shown in Table S8.

***KATP* SNPs and genotype frequencies**

As shown in Table S2, not all 3 *KATP* SNPs examined followed to the Hardy-Weinberg equilibrium (*P*>0.05), only rs1799858 (*P*=0.189) in control subjects did.

**Association of *KATP* SNPs with increased TRIG levels (≥1.7mmol/L) in study subjects**

As shown in Table S3, *KATP* SNPs rs1799858 (adjusted OR=1.45, 95% CI: 0.91-2.31, *P*=0.118), rs4148671 (adjusted OR=1.67, 95% CI: 0.85-3.29, *P*=0.136) and rs78148713 (adjusted OR=1.28, 95% CI: 0.51-3.19, *P*=0.597) were not associated with increased TRIG levels (≥1.7mmol/L).

**Association of *KATP* SNPs with increased TC levels (≥5.2mmol/L) in study participants**

As shown in Table S4, *KATP* SNPs rs1799858 (adjusted OR=1.30, 95% CI: 0.57-2.97, *P*=0.534) and rs4148671 (adjusted OR=0.96, 95% CI: 0.31-2.94, *P*=0.942) were not associated with increased TC (≥5.2mmol/L). In addition, participants with or without increased TC (≥5.2mmol/L) showed significant differences in the distribution of *KATP* SNP rs78148713 (*P*=0.020). However, given that the sample size is relatively small, the potential association between the loci and increased TC may be a biased estimate. Therefore, the OR for the rs78148713 will not be calculated.

**Association of *KATP* SNPs with decreased HDL-C levels (<1.0mmol/L) in study participants**

As shown in Table S5, *KATP* SNPs rs1799858 (adjusted OR=0.99, 95% CI: 0.63-1.57, *P*=0.975), rs4148671 (adjusted OR=0.88, 95% CI: 0.44-1.75, *P*=0.708) and rs78148713 (adjusted OR=0.84, 95% CI: 0.33-2.13 *P*=0.713) were not associated with decreased HDL-C levels (<1.0mmol/L).

**Association of *KATP* SNPs with increased Apo B levels (≥80mg/dL) in study subjects**

As shown in Table S6, *KATP* SNPs rs1799858 (adjusted OR=0.75, 95% CI: 0.49-1.14, *P*=0.174), rs4148671 (adjusted OR=1.09, 95% CI: 0.61-1.98, *P*=0.767) and rs78148713 (adjusted OR=0.62, 95% CI: 0.12-3.15 *P*=0.565) were not associated with increased Apo B levels (≥80mg/dL).

**Association of *KATP* SNPs with decreased Apo AI levels (<120mg/dL) in study participants**

As shown in Table S7, *KATP* SNPs rs1799858 (adjusted OR=0.86, 95% CI: 0.51-1.45, *P*=0.570), rs4148671 (adjusted OR=1.38, 95% CI: 0.60-3.18, *P*=0.450) and rs78148713 (adjusted OR=2.14, 95% CI: 0.68-6.74 *P*=0.195) were not associated with decreased Apo AI levels (<120mg/dL).

**Table legends**

Table S1. The primers of *KATP* SNPs in the SequenomMassARRAY system

Table S2. *KATP* SNPs in study participants

Table S3. Association of *KATP* SNPs with increased TRIG (≥1.7mmol/L) levels in study subjects

Table S4. Association of *KATP* SNPs with increased TC (≥5.2mmol/L) levels in study participants

Table S5. Association of *KATP* SNPs with decreased HDL-C (<1.0mmol/L) levels in study participants

Table S6. Association of *KATP* SNPs with increased Apo B (≥80mg/dL) levels in study subjects

Table S7. Association of *KATP* SNPs with decreased Apo AI (<120mg/dL) levels in study participants

Table S8. Baseline characteristics of study participants at the end of the follow-up

**Table S1. The primers of *KATP* SNPs in the SequenomMassARRAY system**

| **NQ.** | **SNP_ID** | **Gene** | **Protein** | **Primer** | |
| --- | --- | --- | --- | --- | --- |
| 1 | *rs1799858* | ABCC8 | SUR1 | 1st-forward PCR primer (5’-3’) | ACGTTGGATGTGAGGCCCCGACAATCCTCC |
|  |  |  |  | 2nd-reverse PCR primer (5’-3’) | ACGTTGGATGAGTGGGTCCTCACCTCCAAA |
|  |  |  |  | extension primer (5’-3’) | GCCACTCAGGGTTGTGAACCGCAA |
| 2 | *rs4148671* | ABCC9 | SUR2 | 1st-forward PCR primer (5’-3’) | ACGTTGGATGCTTCAAGGATTTATTTCCCC |
|  |  |  |  | 2nd-reverse PCR primer (5’-3’) | ACGTTGGATGAGGTGTAAGTCAAGTAACTC |
|  |  |  |  | extension primer (5’-3’) | GGGGCAAGTAACTCAAGGAAAGATG |
| 3 | *rs78148713* | KCNJ8/ABCC9 | Kir 6.1/SUR2 | 1st-forward PCR primer (5’-3’) | ACGTTGGATGAAGTGGAAGCTGCATGAGAG |
|  |  |  |  | 2nd-reverse PCR primer (5’-3’) | ACGTTGGATGTACTCTTGGGATCTCGGAAC |
|  |  |  |  | extension primer (5’-3’) | CCACTCTTGGGATCTCGGAACAATTTG |

**Table S2. *KATP* SNPs in study participants**

| **NQ** | ***KATP* SNPs** | **MAF in CHB** | **Major/minor allele** | **MAF*** | | ***P_HWE_*-value^#^** | **Power** |
| --- | --- | --- | --- | --- | --- | --- | --- |
|  |  |  |  | **LDL-C <1.8mmol/L** | **LDL-C ≥1.8mmol/L** |  |  |
| 1 | *rs1799858* | 0.165 | *C/T* | 0.181 | 0.212 | 0.189 | 0.998 |
| 2 | *rs4148671* | 0.063 | *C/T* | 0.078 | 0.058 | 0.002 | 0.301 |
| 3 | *rs78148713* | 0.053 | *T/C* | 0.041 | 0.028 | 0.012 | 0.085 |

*MAF: minor allele frequency; CHB：Han Chinese in Beijing, China；^#^*P*_HWE_ value for participants with LDL-C < 1.8mmol/L (control).

**Table S3. Association of *KATP* SNPs with increased TRIG (≥1.7mmol/L) levels in study subjects**

| ***KATP* SNPs** | | **TRIG ≥1.7mmol/L (N/%)** | | **Crude**  **OR (95% CI)** | **Crude**  ***P* value** | **Adjusted**  **OR (95% CI)*** | **Adjusted *P* value*** | **Adjusted**  **OR (95% CI)^#^** | **Adjusted *P*-value^#^** |
| --- | --- | --- | --- | --- | --- | --- | --- | --- | --- |
|  |  | **NO** | **YES** |  |  |  |  |  |  |
| *rs1799858* | *CC* | 279(66.4) | 141(64.1) | 1.00 |  | 1.00 |  | 1.00 |  |
|  | *TT+CT* | 141(33.6) | 79(35.9) | 1.11(0.79-1.56) | 0.554 | 1.21(0.80-1.83) | 0.361 | 1.45(0.91-2.31) | 0.118 |
| *rs4148671* | *CC* | 381(90.7) | 183(83.2) | 1.00 |  | 1.00 |  | 1.00 |  |
|  | *TT+CT* | 39(9.3) | 37(16.8) | 1.98(1.22-3.02) | 0.006 | 1.89(1.03-3.47) | 0.041 | 1.67(0.85-3.29) | 0.136 |
| *rs78148713* | *CC+CT* | 21(5.0) | 11(5.0) | 1.00(0.47-2.11) | 1.000 | 0.81(0.36-1.85) | 0.620 | 1.28(0.51-3.19) | 0.597 |
|  | *TT* | 399(95.0) | 209(95.0) | 1.00 |  | 1.00 |  | 1.00 |  |

*Model 1: After adjustment for gender, age, smoking, drinking, WBC, BMI, EH, T2D, liver function (ALT, AST and Alb), renal function (Scr, BUN and UA), HsCRP, HbA1C, HCY, and RAAS activity (ACE, renin, Ang I, Ang II and ALD)

^#^Model 2a: It is the same as Model 1, also including dyslipidemia (TC, LDL-C, Apo B, HDL-C and Apo AI).

**Table S4. Association of *KATP* SNPs with increased TC (≥5.2mmol/L) levels in study participants**

| ***KATP* SNPs** | | **TC ≥5.2mmol/L (N/%)** | | **Crude**  **OR (95% CI)** | **Crude**  ***P* value** | **Adjusted**  **OR (95% CI)*** | **Adjusted *P* value*** | **Adjusted**  **OR (95% CI)^#^** | **Adjusted *P* value^#^** |
| --- | --- | --- | --- | --- | --- | --- | --- | --- | --- |
|  |  | **NO** | **YES** |  |  |  |  |  |  |
| *rs1799858* | *CC* | 366(66.4) | 54(60.7) | 1.00 |  | 1.00 |  | 1.00 |  |
|  | *TT+CT* | 185(33.6) | 35(39.3) | 1.28(0.81-2.03) | 0.290 | 1.25(0.72-2.17) | 0.431 | 1.30(0.57-2.97) | 0.534 |
| *rs4148671* | *CC* | 486(88.2) | 78(87.6) | 1.00 |  | 1.00 |  | 1.00 |  |
|  | *TT+CT* | 65(11.8) | 11(12.4) | 1.05(0.53-2.09) | 0.879 | 0.78(0.36-1.72) | 0.542 | 0.96(0.31-2.94) | 0.942 |
| *rs78148713* | *CC+CT* | 32(5.8) | 0(0.0) | - | - | - | - | - | - |
|  | *TT* | 519(94.2) | 89(100.0) | - |  | - |  | - |  |

*Model 1: After adjustment for gender, age, smoking, drinking, WBC, BMI, EH, T2D, liver function (ALT, AST and Alb), renal function (Scr, BUN and UA), HsCRP, HbA1C, HCY, and RAAS activity (ACE, renin, Ang I, Ang II and ALD)

^#^Model 2b: It is the same as Model 1, and including dyslipidemia (TRIG, LDL-C, Apo B, HDL-C and Apo AI).

**Table S5. Association of *KATP* SNPs with decreased HDL-C (<1.0mmol/L) levels in study participants**

| ***KATP* SNPs** | | **HDL-C <1.0mmol/L (N/%)** | | **Crude**  **OR (95% CI)** | **Crude**  ***P* value** | **Adjusted**  **OR (95% CI)*** | **Adjusted *P* value*** | **Adjusted**  **OR (95% CI)^#^** | **Adjusted *P* value^#^** |
| --- | --- | --- | --- | --- | --- | --- | --- | --- | --- |
|  |  | **NO** | **YES** |  |  |  |  |  |  |
| *rs1799858* | *CC* | 232(66.3) | 188(64.8) | 1.00 |  | 1.00 |  | 1.00 |  |
|  | *TT+CT* | 118(33.7) | 102(35.2) | 1.07(0.77-1.48) | 0.699 | 0.96(0.65-1.41) | 0.824 | 0.99(0.63-1.57) | 0.975 |
| *rs4148671* | *CC* | 313(89.4) | 251(86.6) | 1.00 |  | 1.00 |  | 1.00 |  |
|  | *TT+CT* | 37(10.6) | 39(13.4) | 1.31(0.81-2.23) | 0.264 | 1.42(0.79-2.53) | 0.242 | 0.88(0.44-1.75) | 0.708 |
| *rs78148713* | *CC+CT* | 18(5.1) | 14(4.8) | 0.94(0.46-1.92) | 0.855 | 1.26(0.56-2.80) | 0.578 | 0.84(0.33-2.13) | 0.713 |
|  | *TT* | 332(94.9) | 276(95.2) | 1.00 |  | 1.00 |  | 1.00 |  |

*Model 1: After adjustment for gender, age, smoking, drinking, WBC, BMI, EH, T2D, liver function (ALT, AST and Alb), renal function (Scr, BUN and UA), HsCRP, HbA1C, HCY, and RAAS activity (ACE, renin, Ang I, Ang II and ALD)

^#^Model 2c: It is the same as Model 1, and including dyslipidemia (TRIG, TC, LDL-C, Apo B, and Apo AI).

**Table S6. Association of *KATP* SNPs with increased Apo B (≥80mg/dL) levels in study subjects**

| ***KATP* SNPs** | | **ApoB ≥80mg/dL (N/%)** | | **Crude**  **OR (95% CI)** | **Crude**  ***P* value** | **Adjusted**  **OR (95% CI)*** | **Adjusted *P* value*** | **Adjusted**  **OR (95% CI)^#^** | **Adjusted *P* value^#^** |
| --- | --- | --- | --- | --- | --- | --- | --- | --- | --- |
|  |  | **NO** | **YES** |  |  |  |  |  |  |
| *rs1799858* | *CC* | 256(64.5) | 164(67.5) | 1.00 |  | 1.00 |  | 1.00 |  |
|  | *TT+CT* | 141(35.5) | 79(32.5) | 0.88(0.62-1.23) | 0.437 | 0.84(0.56-1.24) | 0.366 | 0.75(0.49-1.14) | 0.174 |
| *rs4148671* | *CC* | 354(89.2) | 210(86.4) | 1.00 |  | 1.00 |  | 1.00 |  |
|  | *TT+CT* | 43(10.8) | 33(13.6) | 1.29(0.80-2.10) | 0.298 | 1.36(0.78-2.36) | 0.284 | 1.09(0.61-1.98) | 0.767 |
| *rs78148713* | *CC+CT* | 27(6.8) | 5(2.1) | 0.29(0.11-0.76) | 0.012 | 0.23(0.04-1.28) | 0.094 | 0.62(0.12-3.15) | 0.565 |
|  | *TT* | 370(93.2) | 238(97.9) | 1.00 |  | 1.00 |  | 1.00 |  |

*Model 1: After adjustment for gender, age, smoking, drinking, WBC, BMI, EH, T2D, liver function (ALT, AST and Alb), renal function (Scr, BUN and UA), HsCRP, HbA1C, HCY, and RAAS activity (ACE, renin, Ang I, Ang II and ALD)

^#^Model 2d: It is the same as Model 1, and including dyslipidemia (TRIG, TC, LDL-C, HDL-C and Apo AI).

**Table S7. Association of *KATP* SNPs with decreased Apo AI (<120mg/dL) levels in study participants**

| ***KATP* SNPs** | | **Apo AI <120mg/dL (N/%)** | | **Crude**  **OR (95% CI)** | **Crude**  ***P* value** | **Adjusted**  **OR (95% CI)*** | **Adjusted**  ***P* value*** | **Adjusted**  **OR (95% CI)^#^** | **Adjusted *P* value^#^** |
| --- | --- | --- | --- | --- | --- | --- | --- | --- | --- |
|  |  | **NO** | **YES** |  |  |  |  |  |  |
| *rs1799858* | *CC* | 98(65.3) | 322(65.7) | 1.00 |  | 1.00 |  | 1.00 |  |
|  | *TT+CT* | 52(34.7) | 168(34.3) | 0.98(0.67-1.44) | 0.932 | 0.89(0.56-1.41) | 0.614 | 0.86(0.51-1.45) | 0.570 |
| *rs4148671* | *CC* | 137(91.3) | 427(87.1) | 1.00 |  | 1.00 |  | 1.00 |  |
|  | *TT+CT* | 13(8.7) | 63(12.9) | 1.56(0.83-2.91) | 0.168 | 1.76(0.85-3.65) | 0.127 | 1.38(0.60-3.18) | 0.450 |
| *rs78148713* | *CC+CT* | 6(4.0) | 26(5.3) | 1.35(0.54-3.33) | 0.522 | 2.08(0.75-5.79) | 0.162 | 2.14(0.68-6.74) | 0.195 |
|  | *TT* | 144(96.0) | 464(94.7) | 1.00 |  | 1.00 |  | 1.00 |  |

*Model 1: After adjustment for gender, age, smoking, drinking, WBC, BMI, EH, T2D, liver function (ALT, AST and Alb), renal function (Scr, BUN and UA), HsCRP, HbA1C, HCY, and RAAS activity (ACE, renin, Ang I, Ang II and ALD)

^#^Model 2f: It is the same as Model 1, and including dyslipidemia (TRIG, TC, LDL-C, Apo B, and HDL-C).

**Table S8. Baseline characteristics of study participants at the end of the follow-up**

|  | **LDL-C <1.8mmol/L** | **LDL-C ≥1.8mmol/L** | ***P* value** |
| --- | --- | --- | --- |
| **Sample(N)** | **320** | **320** | **-** |
| **NYHA** |  |  |  |
| I | 140(43.8) | 162(50.6) | 0.053 |
| II | 157(49.1) | 134(41.9) |  |
| III | 21(6.5) | 16(5.0) |  |
| IV | 2(0.6) | 8(2.5) |  |
| **Combined medication** |  |  |  |
| (A)Antiplatelet drugs | 298(93.1) | 300(93.8) | 0.750 |
| (B)Warfarin | 10(3.1) | 17(3.3) | 0.169 |
| (C) Statins | 296(92.5) | 297(92.8) | 0.880 |
| (D) RSIs | 205(66.1) | 221(69.1) | 0.431 |
| (E) BBs | 222(69.4) | 210(65.6) | 0.311 |
| (F) MRA | 67(20.9) | 74(23.1) | 0.504 |
| (G) CCBs | 76(23.8) | 91(28.4) | 0.177 |
| (H) Diuretics | 80(25.0) | 88(27.5) | 0.472 |
| (J) Digoxin | 30(9.4) | 30(9.4) | 1.000 |
| (K) Nitrates | 49(15.3) | 41(14.1) | 0.655 |
| (L) Hypoglycemic agents | 152(47.5) | 179(53.1) | 0.155 |

**References**

1. Mach F, Baigent C, Catapano AL, Koskinas KC, Casula M, Badimon L, Chapman MJ, De Backer GG, Delgado V, Ference BA, et al: **2019 ESC/EAS Guidelines for the management of dyslipidaemias: lipid modification to reduce cardiovascular risk.** *Eur Heart J* 2020, **41:**111-188.

2. Carey RM, Whelton PK, Committee AAHGW: **Prevention, Detection, Evaluation, and Management of High Blood Pressure in Adults: Synopsis of the 2017 American College of Cardiology/American Heart Association Hypertension Guideline.** *Ann Intern Med* 2018, **168:**351-358.

3. Ibanez B, James S, Agewall S, Antunes MJ, Bucciarelli-Ducci C, Bueno H, Caforio ALP, Crea F, Goudevenos JA, Halvorsen S, et al: **2017 ESC Guidelines for the management of acute myocardial infarction in patients presenting with ST-segment elevation: The Task Force for the management of acute myocardial infarction in patients presenting with ST-segment elevation of the European Society of Cardiology (ESC).** *Eur Heart J* 2018, **39:**119-177.

4. Olokoba AB, Obateru OA, Olokoba LB: **Type 2 diabetes mellitus: a review of current trends.** *Oman Med J* 2012, **27:**269-273.

5. January CT, Wann LS, Calkins H, Chen LY, Cigarroa JE, Cleveland JC, Jr., Ellinor PT, Ezekowitz MD, Field ME, Furie KL, et al: **2019 AHA/ACC/HRS Focused Update of the 2014 AHA/ACC/HRS Guideline for the Management of Patients With Atrial Fibrillation: A Report of the American College of Cardiology/American Heart Association Task Force on Clinical Practice Guidelines and the Heart Rhythm Society in Collaboration With the Society of Thoracic Surgeons.** *Circulation* 2019, **140:**e125-e151.

6. Meschia JF, Klaas JP, Brown RD, Jr., Brott TG: **Evaluation and Management of Atherosclerotic Carotid Stenosis.** *Mayo Clin Proc* 2017, **92:**1144-1157.

7. Lang RM, Badano LP, Mor-Avi V, Afilalo J, Armstrong A, Ernande L, Flachskampf FA, Foster E, Goldstein SA, Kuznetsova T, et al: **Recommendations for cardiac chamber quantification by echocardiography in adults: an update from the American Society of Echocardiography and the European Association of Cardiovascular Imaging.** *Eur Heart J Cardiovasc Imaging* 2015, **16:**233-270.

8. Pan Y, Wang T, Li Y, Guan T, Lai Y, Shen Y, Zeyaweiding A, Maimaiti T, Li F, Zhao H, Liu C: **Association of ACE2 polymorphisms with susceptibility to essential hypertension and dyslipidemia in Xinjiang, China.** *Lipids Health Dis* 2018, **17:**241.
